# Evaluating stability of attenuated Sabin and two novel type 2 oral poliovirus vaccines in children

**DOI:** 10.1038/s41541-022-00437-5

**Published:** 2022-02-11

**Authors:** Rahnuma Wahid, Laina Mercer, Chris Gast, Tirza De Leon, Xavier Sáez-Llorens, Alan Fix, Andrew Macadam, Laura Stephens, Konstantin Chumakov, Saskia L. Smits, Marta Murreddu, Jennifer L. Konopka-Anstadt, M. Steven Oberste, Cara C. Burns, Raul Andino, Novilia Sjafri Bachtiar, Erman Tritama, Ananda S. Bandyopadhyay, Gabriela Aguirre, Ricardo Rüttimann, John O. Konz

**Affiliations:** 1 PATH, Seattle, WA USA; 2 Hospital Materno Infantil José Domingo De Obaldía, David, Panama; 3 Centro de Investigación en Vacunas (Cevaxin), Panama City, Panama; 4 Department of Infectious Diseases at Hospital del Niño Dr. José Renán Esquivel, Sistema Nacional de Investigación at Secretaria Nacional de Ciencia y Tecnologia (SENACYT), Panama City, Panama; 5 National Institute for Biological Standards and Control (NIBSC), Hertfordshire, UK; 6 Center for Biologics Evaluation and Research, Food and Drug Administration, Silver Spring, MD USA; 7 Global Virus Network Center of Excellence, Baltimore, MD USA; 8 Viroclinics Biosciences B.V, Rotterdam, the Netherlands; 9 Viroclinics Xplore, Viroclinics Biosciences B.V, Rotterdam, the Netherlands; 10 Division of Viral Diseases, Centers for Disease Control and Prevention, Atlanta, GA USA; 11 Department of Microbiology and Immunology, University of California, San Francisco, CA USA; 12 Research and Development Division, PT. Bio Farma, Bandung, West Java Indonesia; 13 Bill and Melinda Gates Foundation, Seattle, WA USA; 14 Fighting Infectious Diseases in Emerging Countries (FIDEC), Miami, FL USA

**Keywords:** Viral infection, Live attenuated vaccines

## Abstract

Novel oral poliovirus vaccine type 2 (nOPV2) is being developed to reduce the rare occurrence of disease and outbreaks associated with the genetic instability of the Sabin vaccine strains. Children aged 1 to 5 years were enrolled in two related clinical studies to assess safety, immunogenicity, shedding rates and properties of the shed virus following vaccination with nOPV2 (two candidates) versus traditional Sabin OPV type 2 (mOPV2). The anticipated pattern of reversion and increased virulence was observed for shed Sabin-2 virus, as assessed using a mouse model of poliovirus neurovirulence. In contrast, there were significantly reduced odds of mouse paralysis for shed virus for both nOPV2 candidates when compared to shed Sabin-2 virus. Next-generation sequencing of shed viral genomes was consistent with and further supportive of the observed neurovirulence associated with shed Sabin-2 virus, as well as the reduced reversion to virulence of shed candidate viruses. While shed Sabin-2 showed anticipated A481G reversion in the primary attenuation site in domain V in the 5’ untranslated region to be associated with increased mouse paralysis, the stabilized domain V in the candidate viruses did not show polymorphisms consistent with reversion to neurovirulence. The available data from a key target age group for outbreak response confirm the superior genetic and phenotypic stability of shed nOPV2 strains compared to shed Sabin-2 and suggest that nOPV2 should be associated with less paralytic disease and potentially a lower risk of seeding new outbreaks.

## Introduction

Tremendous progress has been made towards the eradication of poliovirus, with both wild type 2 and type 3 polioviruses eradicated and type 1 endemic in only two countries^1,2^. Despite this progress, the spread of circulating type 2 vaccine-derived polioviruses (cVDPV2) represents a major threat to the complete eradication of poliovirus. More specifically, reduced population immunity against the type 2 strain in OPV-using countries following cessation of routine use of type 2 OPV in 2016 precipitated an increase in cVDPV2 circulation, with evidence that targeted responses to cVDPV2 with Sabin OPV type 2 (mOPV2) seeded new outbreaks in adjacent areas^3–5^. The number of acute flaccid paralysis cases associated with cVDPV2 has increased from two in two countries in 2016, to 366 in 16 countries in 2019, and then to over 1000 in 24 countries in 2020 (https://polioeradication.org/polio-today/polio-now).

The underlying genetic mechanism for the evolution of Sabin-2 into VDPV2 is well documented, with key early steps in the loss of attenuation involving reversions in domain IV (U398C) and domain V (A481G) of the internal ribosome entry site within the 5’ untranslated region (UTR) and in amino acid 143 of VP1 capsid protein^6–8^.

Two novel OPV2 candidates have recently been developed and advanced through Phase 1 and 2 clinical studies with the goal of providing similar protection as mOPV2 but with reduced risk of loss of attenuation^9–11^. Both candidates include a genetically stabilized domain V termed S15, which was designed to attenuate the strain to a similar degree as the Sabin strains, while avoiding the possibility of loss of attenuation via single nucleotide changes. More specifically, the potential for thermodynamic strengthening of the RNA structure via point mutations was reduced by eliminating the use of U-G base pairs^12^.

In addition to the S15 domain V, novel OPV2 candidate 1 (nOPV2-c1) also includes relocation of the cis-acting replication element (cre) to the 5’ untranslated region (UTR) of the virus in order to protect the S15 domain V from replacement through a single recombination event. In addition, the nOPV2-c1 includes two modifications to the 3D polymerase which reduce the frequency of recombination events and improve replication fidelity^13^. The novel OPV2 candidate 2 (nOPV2-c2) supplements the S15 domain V with codon deoptimization of the capsid region to further attenuate the strain^14^.

Both candidates were shown to be generally safe and well tolerated in Phase 1 and 2 clinical studies^10,11,15^. In infants, nOPV2-c1 induced non-inferior seroprotection rates compared to Sabin OPV2 at both 10^5^ and 10^6^ CCID_50_ (50% cell culture infectious dose) doses, while nOPV2-c2 met the non-inferiority criterion at the 10^6^ CCID_50_ dose but not at the 10^5^ CCID_50_ dose^11^.

In addition to having an acceptable clinical safety profile and non-inferior immunogenicity, the novel vaccines are intended to retain their attenuated phenotype in a manner superior to Sabin OPV2. Details of a workflow to examine the genetic heterogeneity and neurovirulence of fecally-shed virus using next-generation sequencing and mouse neurovirulence assay have recently been described^9^. Here, this paper reports the first use of this workflow to provide a head-to-head comparison of shed nOPV2 candidates with shed Sabin-2 from two clinical trials in Panamanian children.

## Results

Samples for evaluation of genetic stability of shed virus following administration of monovalent OPV2 (mOPV2) or nOPV2 candidates were collected from 1-to-5-year-old children in Panama during phase 4 and phase 2 trials (M2 and M5, described in METHODS). A modified transgenic mouse neurovirulence test (mTgmNVT) was utilized to confirm whether the attenuation phenotype of nOPV2 candidates was maintained in shed virus. In addition, to assess the genetic stability of the genome in shed virus, next-generation sequencing (NGS) was conducted. NGS and mTgmNVT were used to evaluate virus shed in a subset of stool samples (the exploratory endpoint specimen, EES) from clinical trial participants, as described in METHODS^9^.

The M2 and M5 studies were conducted non-contemporaneously, with the global cessation of OPV2 usage in routine immunization occurring between the two study periods. As such, due to differences in prior polio vaccination regimens, children in the included age group had varying levels of baseline intestinal immunity. As shown in Table 1, the number of EES identified differs between M2 and M5 cohorts: 6 EES from children administered mOPV2 and >30 EES each from the nOPV2-c1 and nOPV2-c2 cohorts. This is because 90% of the children in M2 had prior trivalent OPV (tOPV) exposure, leading to reduced fecal shedding and thereby a large fraction of M2 participants never shed ≥4 log CCID_50_/g stool^11^, the threshold for EES selection. Notably, the vast majority of 1-to-5-year-old participants in the M5 trial were previously vaccinated with IPV and/or bOPV, and only 10% had received tOPV.

**Table 1:** Sample availability (post dose1) from children and genetic stability analyses performed.

	M2 mOPV2	M5 nOPV2-c1	M5 nOPV2-c2
Vaccinated participants	50	49	51
Participants with prior tOPV exposure	45	6	4
EES day range	4–28	2–28	1–21
Number of EES	6	31	38
EES evaluated by NGS	6	31	38
Number of isolates available for NV^1^	5	30	37
Number of EES with NV results available	5	22	29

^1^Virus from one mOPV2 (D28) and one nOPV2-c1 (D5) EES failed to amplify adequately in cell culture and could not be tested. nOPV2-c2 D1 EES positive for type 2 by PCR, but only 1 stool replicate produced usable NGS data. Stool 2 and isolate did not produce usable NGS data. Isolate shown by PCR to be enterovirus positive. This EES was removed from mTgmNVT pipeline.

### Neurovirulence of shed virus

As shown in Table 1, mTgmNVT results are available for a majority of the EES. Invalid tests were not repeated as the genetic characteristics of the EES without a mTgmNVT result were not unique, having also been featured in other EES that had valid mTgmNVT results.

**Fig. 1:**
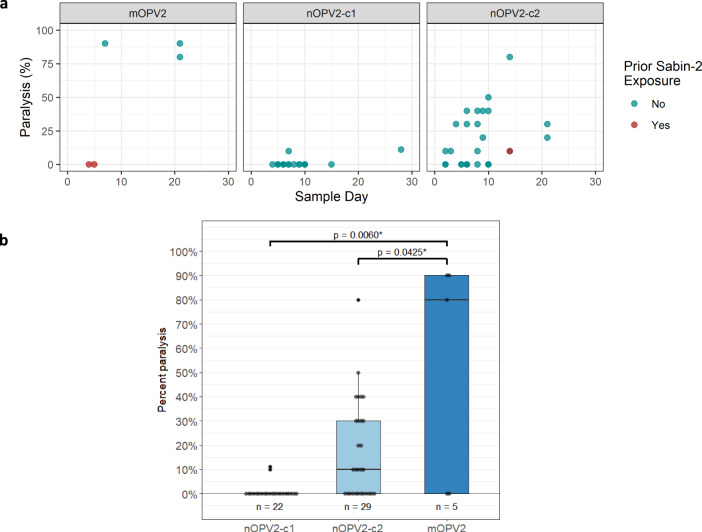
Neurovirulence summary. **a** Measured paralysis rate in modified mouse neurovirulence test. Datapoints are percent paralysis per EES. Paralysis rates for shed virus samples are indicated versus day of EES per vaccine. EES from participants with and without prior Sabin-2 exposure (via routine immunization) are shown by red or green circles. **b** Comparative analysis of neurovirulence. Datapoints are percent paralysis per EES following intraspinal administration of 10^4^ CCID_50_ virus inoculum per mouse in the mTgmNVT. The *p* values indicated are based on estimated odds ratio of paralysis for shed virus of each nOPV2 candidate compared to Sabin-2. Displayed are the median (horizontal line), first and third quartiles (lower and upper box boundaries, respectively), with whiskers extending to the largest (smallest) value within 1.5 times the inter-quartile range of the upper (lower) box boundary.

Descriptive (Fig. 1a) and comparative (Fig. 1b) analyses of the test data were performed to determine the virulence of shed nOPV2 and Sabin-2 viruses, as well as to make a statistical comparison of shed nOPV2 to shed Sabin-2, where possible.

For mOPV2, five EES (one per participant, ranging from days 2 to 21 post dose 1) were tested (Fig. 1a). Two EES from days 4 and 5, which were from participants with tOPV vaccination history, showed no paralysis. Three EES from day 7 and 21 from participants with no prior documented OPV2 exposure showed high paralysis rates. Although only a few EES were available from mOPV2- administered participants, the data demonstrate that Sabin-2 reverts as expected and, when reverted, causes high paralysis rates in the mouse model, ranging up to 90%. Descriptive model-based analysis indicates the absence of a detectable mouse gender effect (*p* = 1.0000), with estimated mean paralysis rate (across both mouse genders) of 32.2% (95% CI 0.003%, 98.6%).

For nOPV2-c1, 22 EES (one per participant, ranging from days 4 to 28 post dose 1 vaccination) had valid results, with two EES paralyzing a single mouse each and no paralysis for the remaining 20 EES (range 0 to 11.1%). Descriptive model-based analysis indicates the absence of a detectable mouse gender effect (*p* = 0.9827), with estimated mean paralysis rate of 0.03% (95% CI < 0.1%, 26.7%).

Similarly, for nOPV2-c2, 29 EES (one per participant ranging from days 2 to 21) had valid results. Paralysis rates ranged from 0 to 80%, with six EES paralyzing ≥ 40% of mice (discussed further under Next-generation sequencing of shed virus). Descriptive model-based analysis indicates lower paralysis rates for female versus male mice (*p* = 0.0023), with overall estimated mean paralysis rate of 8.8% (95% CI 3.9%, 18.9%).

Results of the comparative model-based analysis indicate the absence of a detectable mouse gender effect (*p* = 0.9789), and significantly reduced odds of mouse paralysis from virus obtained from nOPV2-c1 recipients compared to mOPV2 recipients (adjusted odds ratio [aOR] = 0.001, 95% CI < 0.001, 0.121, *p* = 0.0060) (Fig. 1b). There is a detectable mouse gender effect (*p* = 0.0031, with odds of paralysis of female mice reduced relative to male mice), and a significant (*p* = 0.0425) reduction in the estimated odds of mouse paralysis from virus obtained from nOPV2-c2 recipients compared to mOPV2 recipients (aOR = 0.097, 95% CI 0.010, 0.919).

### Next-generation sequencing of shed virus

NGS was performed on full-length poliovirus genome following PCR amplification of virus present in stool and cell culture-amplified virus from the selected stool samples. Descriptive analysis of the frequency of polymorphisms observed by NGS in key attenuating regions of Sabin-2 (398, 481, and VP1-143) or attenuating and modified regions of the nOPV2 shed virus was conducted to assess retention of the engineered modifications, and determine if the sequence variations in shed virus correlate with the observed neurovirulence results in the transgenic mouse model.

In addition, coverage maps were used to determine if any co-infection (and potential recombination) was observed in the EES. Following NGS, the sample reads were mapped to a type 2 reference (Sabin-2 or candidate 1 or 2, as appropriate) as well as to Sabin 1 and 3 references. Non-polio enteroviruses, Sabin-1, and Sabin-3 were not detected in any of the EES evaluated by NGS and no recombinant viruses were observed.

**Fig. 2:**
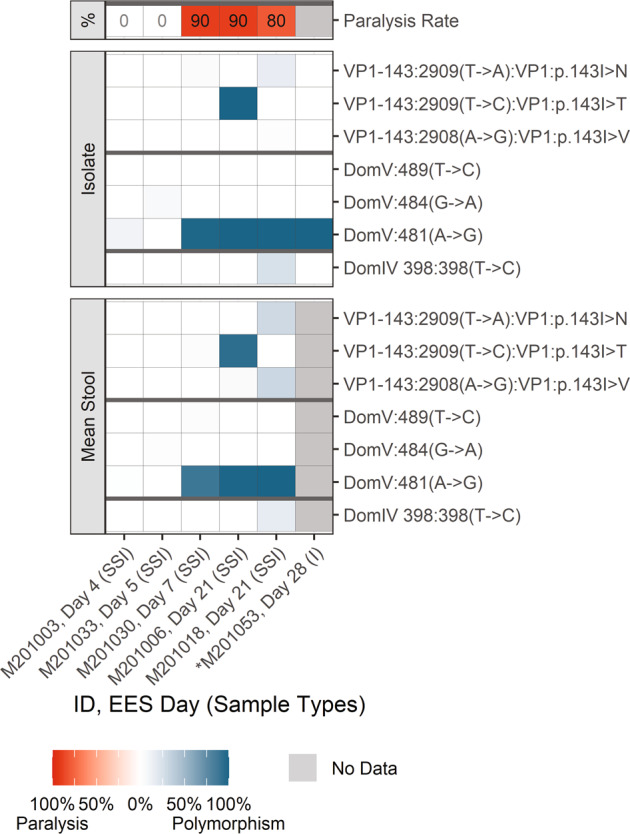
mOPV2. Frequency of polymorphisms in EES at known attenuation sites and mTgmNVT results. EES day shown with mTgmNVT result (red color gradient) and polymorphism frequency (blue color gradient) averaged across two stool replicates (SS) and culture-amplified virus (I), if present. Amino acid associated with SNP indicated, if applicable. S = Stool replicate; I = Culture-amplified virus isolate (SSI = Two stool replicates and culture-amplified virus isolate; SI = Single stool replicate and isolate). White cells = polymorphism not detected. Gray cells = mTgmNVT result not available or no NGS data from stool (S)/ culture-amplified virus (I). *Day 28 = Insufficient titer to test EES in mTgmNVT. The NGS pipeline reports SNPs. Coding impact assumes changes are not in common genomes when multiple polymorphisms are observed in VP1-143. All SNPS reported have Quality scores (Q scores) ≥ 30.

As expected, shed Sabin-2 virus reverted rapidly (by day 7). NGS data (Fig. 2 and Supplementary Table 1) support the high levels of virulence observed in the mTgmNVT for the three EES from Days 7 and 21 post-vaccination, with a fixed reversion of the primary attenuation site in domain V (A481G) in these samples. EES from earlier collection days do not show 481G reversion or paralysis. Reversion of VP1-143 and/or position 398 in day 7 and later samples was observed. Several polymorphisms at variable frequency were observed in other regions of the genome. Only those mutations leading to amino acid changes and meeting reporting criteria are summarized (Supplementary Table 2).

NGS was performed directly on virus in stool (two replicates of each EES stool) and on virus cell-culture amplified from stool, where available, for 31 nOPV2-c1 participants. Polymorphisms noted in stool were also largely observed in the culture-amplified virus (used in the mTgmNVT), and there was strong correlation between the single nucleotide polymorphisms (SNPs) observed directly in stool versus amplified virus, demonstrating minimal bias from the cell-culture amplification process. Supplementary Fig. 1 shows the correlation data for SNPs relevant to virulence and viral fitness.

**Fig. 3:**
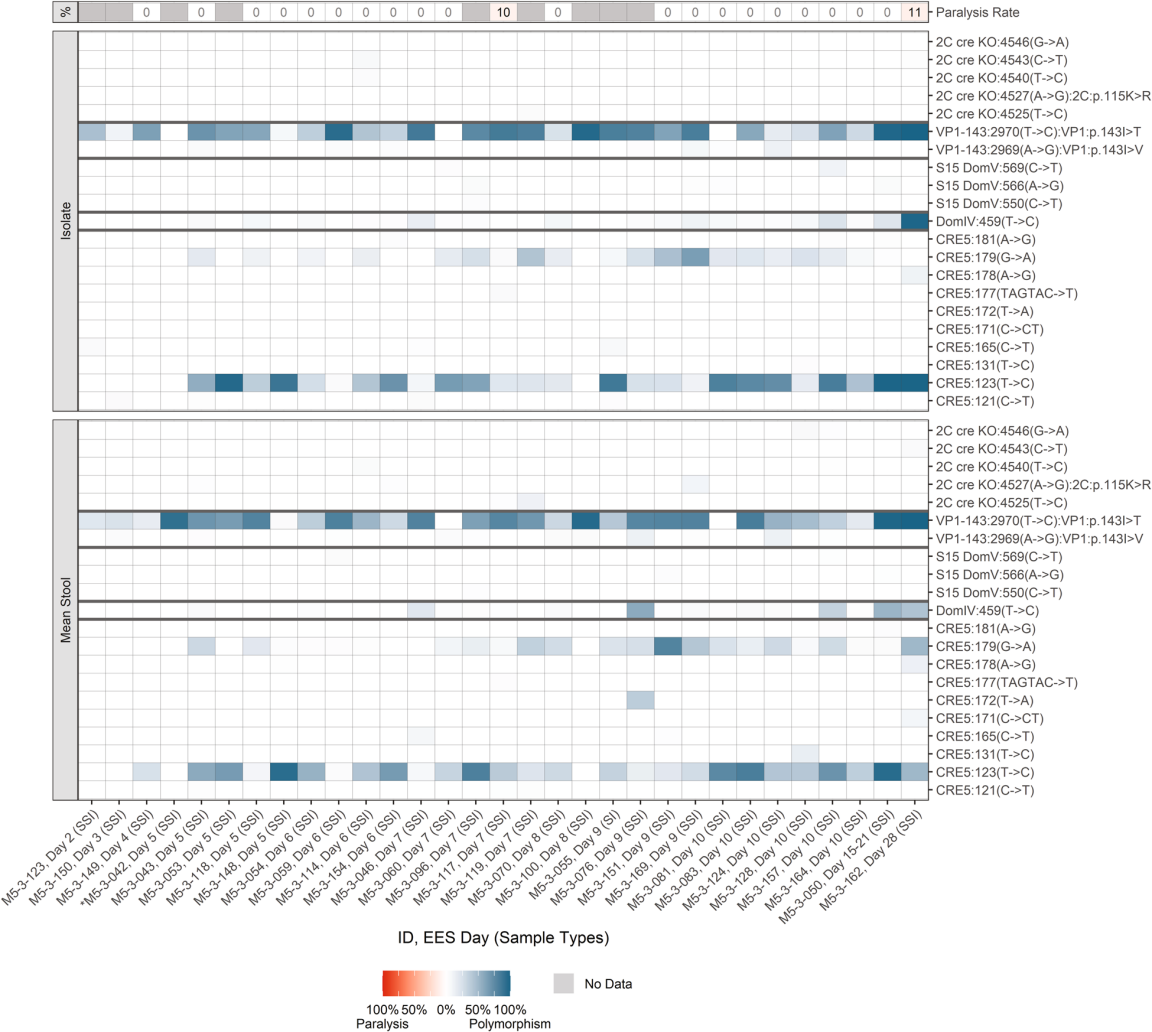
nOPV2-c1. Frequency of polymorphisms in EES at known attenuation sites and mTgmNVT results. EES day shown with mTgmNVT result (red color gradient) and polymorphism frequency (blue color gradient) averaged across two stool replicates (SS) and culture-amplified virus (I), if present. Amino acid associated with SNP indicated, if applicable. S = Stool replicate; I = Culture-amplified virus isolate (SSI = Two stool replicates and culture-amplified virus isolate; SI = Single stool replicate and isolate). White cells = polymorphism not detected. Gray cells = mTgmNVT result not available or no NGS data from stool (S)/ culture-amplified virus (I). *Day 5 = Insufficient titer to test EES in mTgmNVT. The NGS pipeline reports SNPs. Coding impact assumes changes are not in common genomes when multiple polymorphisms are observed in VP1-143. All SNPS reported have Q scores ≥ 30.

Mutations in the modified regions and known attenuating mutations are summarized in Fig. 3 and Supplementary Table 3 for nOPV2-c1. Evolution of the shed virus was consistent with observations from prior studies in adult recipients of nOPV2-c1^9^. SNPs in the relocated cre at nucleotide 123 and/or 179, which strengthens U-G base pair to C-G or U-A were observed in all EES day 4 to 28 at variable levels up to 100% frequency. Several other SNPs were observed in the relocated cre region at low levels, although U172A was observed at higher levels in the stools of a day 9 EES. In addition, several samples accumulated C121U and/or A181G polymorphisms at <10% average frequency in samples when present. These polymorphisms allow an additional base pair at 121-181 through formation of U-A or C-G pair, likely extending the stem length of cre5.

Reversion in domain V (nucleotides 468-535) of the 5’ UTR is the main determinant for restoration of virulence (as measured in the mouse neurovirulence model) following mOPV2 administration in humans. No polymorphisms consistent with increased virulence were detected in S15 domain V of shed nOPV2-c1. Although several SNPs were observed at low levels (often unconfirmed by replicates and always ≤10% in any replicate), none are pair-forming or pair-strengthening. Within the 5’ UTR, variation was also noted at position 459 in domain IV, corresponding to nucleotide 398 in Sabin-2, which is known to have minor impact on attenuation (modest increase in neurovirulence).

Mutations that result in reversion of the unprotected secondary attenuation site, VP1-143 (e.g. T2970C coding for VP1-143T), were observed consistently in the EES, often at consensus or higher levels.

No mutations impacting the modified Rec1 or Hifi3 locations of the 3D polymerase were observed, but a few low-level silent polymorphisms in the cre knockout region were observed (e.g., U4540C). One mutation in the cre KO regions was associated with an amino acid change, although it was observed only in single replicates of the day 5, 6 and 9 EES.

Two samples with limited paralysis (one mouse each) had high levels of VP1-I143T and some strengthening of the cre 123/179 pair; one also had U459C reversion (day 28 EES had fixed changes at 123/179, 459 and VP1-143 had a measured paralysis rate of 11%). These polymorphisms were previously shown to have an additive impact on neurovirulence in the mouse model^9,13^.

**Fig. 4:**
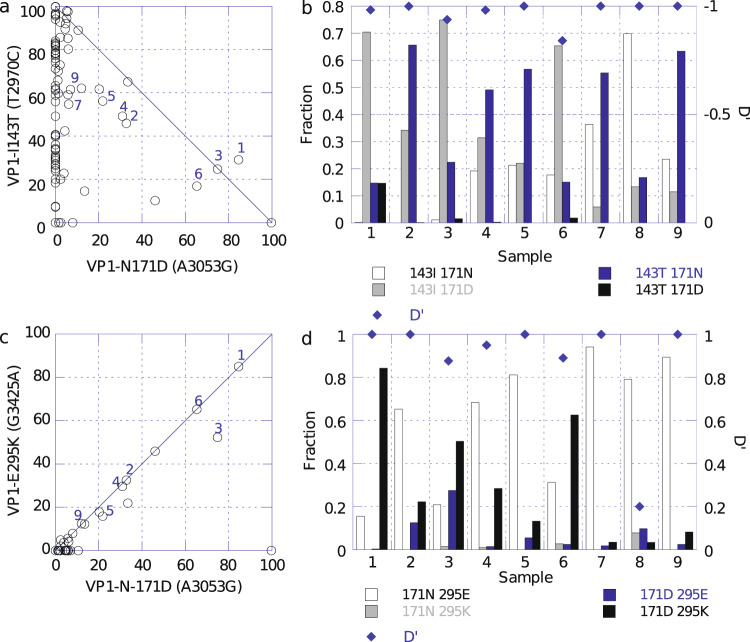
Relationship between VP1-I143T, VP1-N171D and VP1-E295K in nOPV2-c1 shed virus samples. **a** Levels of T2970C (VP1-I143T) and G3053A (VP1-N171D) in individual replicates from 31 EES. Numbered symbols correspond to the sample number in panel **b**. **b** Colocation of 143T and 171D in nine samples from three study participants. **c** Levels of A3053G (VP1-N171D) and G3425A (VP1-E295K) in individual replicates from 31 EES. Numbered symbols correspond to the sample number in panel **d**. **d** Colocation analysis for 171D and 295K in the same nine samples as panel **b**.

Several SNPs at variable frequency were observed in other regions of the genome. Mutations associated with amino acid changes and meeting reporting requirements are summarized in Supplementary Table 4. Some of these (VP3-E234K, VP1-N171D and VP1-E295K) are present at moderate levels in the vaccine lots and thus are expected to appear in shed virus^16^. For example, the level of VP3-E234K in the lot used in this study was 45%. As anticipated from research studies on molecular clones containing this mutation, there was no consistent pressure for or against this mutation. VP1-E295K (G3425A) frequencies were nearly always less than or equal to VP1-N171D (A3053G), and they are often present at similar levels in the EES (Fig. 4c). To determine whether these mutations were present on the same genome, a co-location analysis was performed on a subset of samples evaluating paired NGS sequence reads which covered the sequence from 3053 to 3425 (Fig. 4d). This evaluation showed that these two mutations are predominantly co-located. Relative linkage disequilibrium constants^17^ (D’ in Fig. 4d) also were calculated and confirmed the positive association. Conversely, using the same approach for reads including 2970 and 3053, variants in which VP1-I143T and VP1-N171D are co-located, were shown to be disfavored in vaccine bulk and also appear not to be selected during replication in the human gut (Fig. 4a, b). Total frequencies for the two variations are rarely above 100% and co-location analysis of the same subset of samples shows a double mutant at >2% in only one of the 9 samples. Relative linkage disequilibrium measures confirm the negative association.

**Fig. 5:**
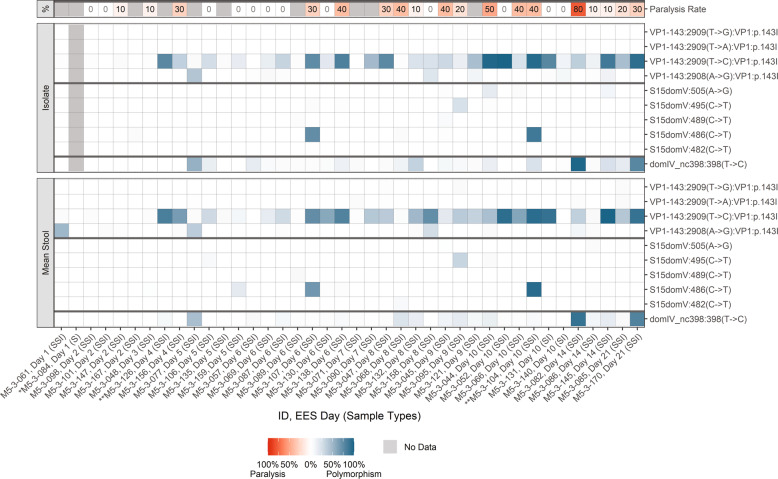
nOPV2-c2. Frequency of polymorphisms in EES at known attenuation sites and mTgmNVT results. EES day shown with mTgmNVT result (red color gradient) and variant frequency (blue color gradient) averaged across two stool replicates (SS) and culture-amplified virus (I), if present. Amino acid associated with SNP indicated, if applicable. S = Stool replicate; I = Culture-amplified virus isolate (SSI = Two stool replicates and culture-amplified virus isolate; SI = Single stool replicate and isolate). White cells = polymorphism not detected. Gray cells = mTgmNVT result not available or no NGS data from stool (S)/ culture-amplified virus (I). *Day 1 = Insufficient titer to test EES in mTgmNVT The NGS pipeline reports SNPs. Coding impact assumes changes are not in common genomes when multiple polymorphisms are observed in VP1-143. All SNPs reported have Q scores ≥ 30.

NGS was also performed on stool (two replicates of each EES stool) and culture-amplified virus where available for 38 participants who received nOPV2-c2. Polymorphisms in the modified regions and known attenuating sites are summarized in Fig. 5 and Supplementary Table 5.

As shown in Fig. 5, the mutation in domain IV (U398C, equivalent to U459C in nOPV2-c1) and reversion of VP1-143 (primarily associated with an amino acid change from isoleucine to threonine) was observed in most nOPV2-c2 samples. U398C was observed at 98% in a day 14 EES along with partial reversion at VP1-143 and is associated with a paralysis rate of 80%.

Five polymorphisms were observed in domain V (described further below in S15 Domain V polymorphisms), although none are known to be associated with reversion to virulence. C486U at consensus level was observed in a day 6 EES. While not possible to assess for co-location, levels are very similar to U2909C mutation yielding VP1-I143T (73% versus 75%) suggesting the possibility that the domain V polymorphism is a carrier mutation selected due to co-location on a genome with VP1-143T.

There were no mutations in the modified CpG sites in the P1 region meeting the reporting algorithm described in METHODS, although several changes were observed in the other regions of the genome (Supplementary Table 6). A known Vero cell adaptation present in vaccine bulk, VP4-A41V, is also observed in shed virus. VP3-G212S is also present in vaccine bulk and is selected in a few of the stools. However, there is no apparent relationship of this mutation to virulence. Similarly, mutations in 3C and 3D polymerase were observed in a minority of samples, but again not associated with higher neurovirulence in the mTgmNVT.

To further characterize the virulence of the shed nOPV2-c2 viruses, the 6 EES with paralysis rate ≥40% were evaluated in multi-dose neurovirulence tests to determine the 50% paralytic dose (PD_50_) compared to nOPV2-c2 (Table 2). In general, the more virulent samples (lower PD50) contain higher levels of U398C and VP1-I143V/T variants (e.g., day 14 EES with fixed U398C and partial reversion at VP1-143 has a calculated PD_50_ of 3.86).

**Table 2:** Paralytic dose 50 (PD50) and key polymorphisms in culture-amplified shed nOPV2-c2 virus with higher virulence.

Subject ID	EES Day	Paralysis (%), 4 log CCID_50_	PD_50_^1^, log CCID_50_ (CI)	U398C, % in isolate	Domain V SNPs in	VP1-143 total, % in isolate
nOPV2-c2	NA	0	6.5 (6.13, 6.87)	<1%	<1%	<1%
M5-3-138	6	40	4.57 (3.93, 5.20)	13	1% C495U	81
M5-3-068	8	40	>4.8 (1/9)^2^	11	3% C486U	1
M5-3-045	9	40	4.44 (4.05, 4.82)	8	2% C486U, 6% C489U 1% A505G,	30
M5-3-066	10	40	>5.1 (0/10)^2^	0	3% A505G	24
M5-3-044	10	50	4.71 (4.19, 5.23)	14	1% C495U, 16% A505G	98
M5-3-082	14	80	4.03 (3.50, 4.57)	98	—	37

^1^Virulence of viruses was determined in Tg PVR21 mice using intraspinal inoculation.

^2^PD50 inestimable. Proportion of mice paralyzed at highest dose are indicated.

### S15 Domain V polymorphisms

Sequence analysis of EES from both nOPV2 candidates revealed no variants in any of the U-A base-pairs that were originally introduced into the S15 domain V to determine and stabilize the attenuation phenotype^12^.

**Fig. 6:**
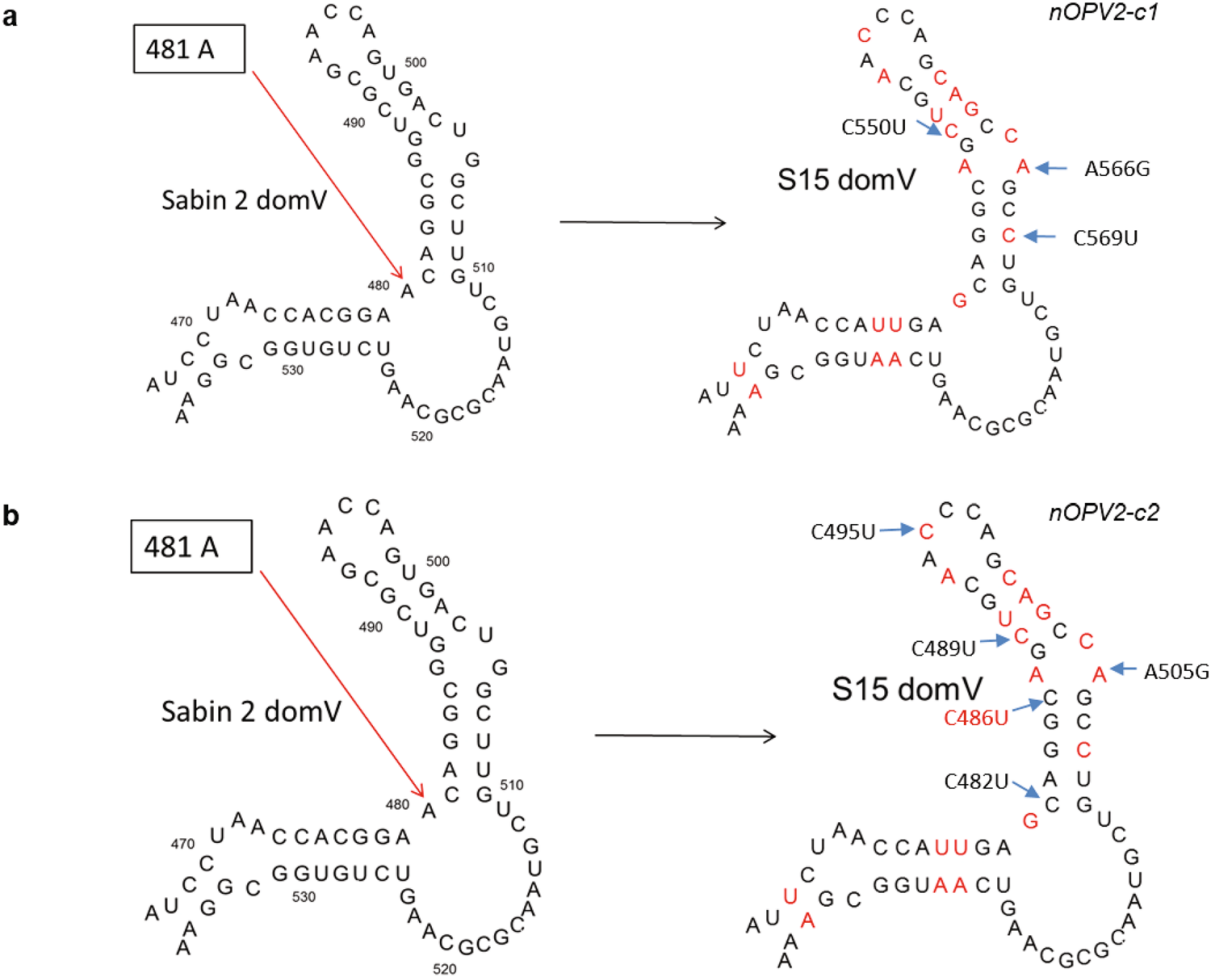
Polymorphisms in predicted RNA secondary structure of S15 domain V. SNPs indicated by arrows. Polymorphism in red is present at high frequency in EES. Polymorphisms in black are present at variable frequency, but confirmed in replicates of the EES. **a** nOPV2-c1. **b** nOPV2-c2.

In the nOPV2-c1 EES (Fig. 6a), C550U was observed at 2% in one sample and C569U in 3 samples at a maximum frequency of 6% in a single replicate of stool. These two SNPs appear to destabilize C-G base pairs. A566G, observed in 5 EES at ≤4% frequency, is in a loop structure of domain V, is polymorphic in enteroviruses^18^, and is anticipated to have no impact (also supported by RNA structure prediction model showing similar energy structure for S15 domain V with and without this SNP). This is in contrast to four of the six nucleotides in this loop that are 100% conserved.

For nOPV2-c2, 5 SNPs were observed in domain V (Fig. 6b). C482U is observed in one replicate of a day 8 EES at 12% frequency. This appears to destabilize a C-G base pair. Similarly, C486U discussed earlier was observed in 11 EES and also appears destabilizing (C-G to U-G) as supported by RNA structure modeling, which predicts a less stable structure. C489U was observed in 3 samples and is in the same position as C550U in nOPV2-c1. It has a similar destabilizing impact on the structure. A505G was observed in 6 nOPV2-c2 samples and is present in a loop of domain V in the same position as 566 of nOPV2-c1, and has a similar lack of impact on the predicted energy of the RNA structure. C495U, also present within a loop, was observed in 5 samples and shows a similar lack of impact on predicted stability of the domain V.

## Discussion

The most consequential safety risks associated with use of mOPV2 are rare cases of vaccine-associated paralytic poliomyelitis (VAPP) in vaccine recipients or their close contacts, and reversion and circulation of the vaccine strain in the community with emergence of vaccine-derived polioviruses in settings of persistently low immunization rates. A primary objective of nOPV2 development is to substantially reduce the potential for reversion to virulence, if not eliminate the risks posed by mOPV2. To address the rapidity and extent to which nOPV2 strains and Sabin-2 reacquire virulence in vaccinated children, their genetic stability was assessed using next-generation sequencing, and phenotypic stability was assessed using a mouse neurovirulence test on shed vaccine virus samples.

As expected, Sabin-2 virus reverted rapidly, with shed virus showing known key genetic reversions soon after vaccination and corresponding high paralysis rates in the mouse model once reverted. Although the number of samples available was small, the results were consistent with historic data for excreted Sabin-2 viruses^7,19^ and molecular clones with relevant mutations (e.g. A481G). In contrast, no changes that would be expected to contribute to loss of attenuation were observed in S15 domain V of the shed nOPV2 strains. Generally, although SNPs were observed in shed nOPV2 strains, they were at low levels and none led to strengthening of a base pair. The specific observed changes generally appeared to be neutral or destabilizing (i.e., they do not appear to contribute to thermodynamic strengthening of the RNA structure) and theoretically further attenuate the shed nOPV2 viruses.

The most noteworthy genetic changes observed in nOPV2 strains were mutations leading to (a) amino acid substitutions at VP1-143, a U398C polymorphism (in Sabin-2 and nOPV2-c2; U459C in nOPV2-c1) in domain IV and (b), polymorphisms that strengthen a base pair in the relocated cis-acting replication element (cre) or extend the relocated cre in nOPV2-c1. These are potentially relevant to neurovirulence and viral fitness, though largely anticipated from prior work studying the evolution of the Sabin-2 and nOPV2 strains. Some mutations previously identified in the nOPV2-c1 vaccine lots and characterized phenotypically^16^ showed anticipated behavior upon replication in vivo. For example, VP1-E295K—which causes a temperature-sensitive defect limiting replication at 37 °C—was selected against unless compensated for by the presence of a VP1-N171D mutation. VP1-I143X and VP1-N171D mutations, which increase mouse neurovirulence individually but not additively, were only exceptionally selected together in the same virus.

The results from the mouse neurovirulence tests indicated a statistically significant reduction of the paralysis rate for shed virus from nOPV2-c1 or nOPV2-c2 recipients relative to mOPV2 recipients, and are consistent with prior preclinical and clinical data^13,14^. For shed nOPV2-c2 EES with paralysis rates of ≥40%, the multi-dose mouse assay provided PD_50_ estimates consistent with previous data using molecular constructs^9^. These showed that U398C or VP1-143V/T in the nOPV2-c2 clone contribute to virulence in the mouse neurovirulence model (PD_50_ 4.0 and 3.9, respectively) using Tg66 mice. Combining the two mutations in one clone resulted in a more virulent virus (PD_50_ 2.4), indicating an additive effect. Shed virus from all 6 EES show higher virulence than nOPV2-c2 (PD_50_ 6.3) and Sabin-2 (PD_50_ 5.9), although all are still considerably less virulent than Sabin-2 with reversion at 481 in domain V (PD_50_ 1.8).

The results described here suggest that nOPV2-c1 may increase moderately in fitness and virulence over several weeks replication in the human gut through mutations in the cre5 (U123C/G179A), U459C, VP1-143 and similarly for nOVP2-c2 via U398C and VP1-143. This contrasts with the rapidity of reversion of Sabin-2, whereby highly virulent viruses are selected in all recipients excreting virus longer than 7 days after vaccination. No mechanism for further increases in virulence of nOPV2 is apparent from these results. Recombination with Sabin or *Enterovirus* C viruses could potentially yield more virulent viruses, but nOPV2 contains modifications aimed at reducing the chances of this happening. Indeed, despite occasional detection of Sabin-1 or −3 by Sabin quadplex rRT-PCR, there was no evidence of nOPV2 recombinant viruses in these samples. Recombination will be evaluated more comprehensively in an analysis of the larger infant cohorts, a separate arm of these same M2 and M5 clinical trials^11^.

**Fig. 7:**
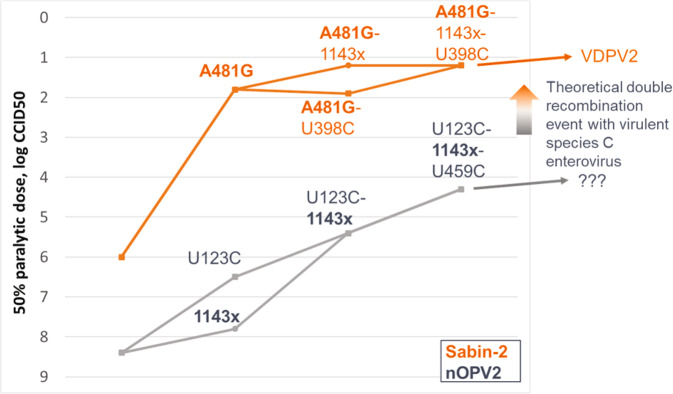
Framework of genetic evolution of Sabin-2 and nOPV2-c1 in humans and impact on neurovirulence in Tg66 mice. 1143x refers to mutations in VP1-143 (isoleucine to threonine or valine).

A framework (Fig. 7) linking the genetic and phenotypic evolution is provided, which suggests that neurovirulence—as measured using paralysis in the transgenic mouse model—is unlikely to approach the levels seen for reverted Sabin-2 unless the genetically-stabilized S15 domain V is replaced. As for nOPV2-c1, the overall conclusion is that nOPV2-c2 is also considerably less likely than Sabin-2 to evolve towards significant virulence in the time span studied here, which generally captures the extent of duration of viral shedding of infectious quantities of virus.

Although there is no direct way to quantitatively extrapolate to reduced risk of paralysis in humans, the available data support the superior genetic and phenotypic stability of shed nOPV2 compared to shed Sabin-2. Furthermore, the data suggest that shed nOPV2 does not (and likely cannot) evolve through single site mutation and selection alone to the level of virulence observed for shed Sabin-2 virus. It is, therefore, likely that the lower/limited evolution of nOPV2 could translate to a lower risk of VAPP relative to mOPV2. Similarly, there is also reduced likelihood of nOPV2 evolving to virulent cVDPV strains following circulation in under-immunized populations.

Vaccination with nOPV2 in outbreak settings features prominently in the Global Polio Eradication Initiative’s new strategy to stop further spread of cVDPV2^20^. As a result of the safety, immunogenicity and promising phenotypic stability of these viruses, Emergency Use Listing (EUL) has recently been granted by WHO for nOPV2-c1 (https://www.who.int/news/item/13-11-2020-first-ever-vaccine-listed-under-who-emergency-use). Initial use of this vaccine under EUL has begun and enhanced pharmacovigilance and environmental surveillance have been implemented to further evaluate this strain under real-world conditions.

## Methods

### Novel OPV2 candidates

The nOPV2 vaccine candidates are attenuated serotype 2 polioviruses derived from a modified Sabin type-2 infectious cDNA clone. The two candidates (nOPV2-c1, nOPV2-c2) both include the previously described S15 genetic stabilization of domain V in the 5’ UTR^12^. Briefly, nOPV2-c1 also includes relocation and modification of the cis-acting replication element (cre) to the 5’ end of the genome, inactivation of cre element in the 2C genomic region by introducing silent mutations, and modifications of the 3D polymerase protein to increase fidelity and reduce recombination^13^. In addition to the domain V modification, nOPV2-c2 includes synonymous codon deoptimization of the capsid (P1) region, with approximately 40% of the available sites modified through incorporation of CpG dinucleotides in the second and third positions of the codons^14^.

Production lots of nOPV2-c1 and nOPV2-c2 were manufactured and released by P.T. Bio Farma (Bandung, Indonesia).

### Phase 4 clinical trial with mOPV2

In advance of the global type 2 oral poliovirus vaccine cessation in 2016, a Phase 4 trial (M2 study, NCT02521974) was conducted in which mOPV2 (dose of ≥10^5^ CCID_50_, Polio Sabin Mono Two, GlaxoSmithKline Biologicals, Belgium) was administered to 50 healthy 1-to-5-year-old Panamanian children who had previously received trivalent OPV (tOPV) or IPV vaccinations^21^. The protocol was approved by the Ethical Review Committee of the Hospital del Niño 144 “Dr. José Renán Esquivel”. Written consent was obtained from parents or legal guardian(s) at enrollment per country regulations. Trial participants were randomized to receive either one or two doses of mOPV2, with the second dose occurring 4 weeks after the first. Stool samples were collected daily until day 10 following each vaccination, and then on days 14, 21, and 28.

Daily stool samples were collected and periodically shipped to the Centers for Disease Control and Prevention (CDC, Atlanta, GA, USA), where they were stored at −20 °C until analysis. Type-2 poliovirus genomes were detected using a Sabin multiplex real-time RT-PCR (rRT-PCR) assay of total nucleic acid extracted from stool suspensions (50%, w/v)^22^. In samples positive for type-2 poliovirus, infectious virus was titrated in culture and measured as CCID_50_/g stool by a modification of the WHO cell sensitivity assay^23^. For each participant, the last collected sample post dose 1 with at least 4.00 log_10_ CCID_50_ per gram of stool was termed the EES (exploratory endpoint specimen).

Stool suspensions (10% w/v in EMEM, Gibco) of the EES were prepared for next-generation sequencing (NGS). To achieve the required concentration of virus for mouse neurovirulence assays, virus was amplified in HEp-2C cells (ATCC, CCL-23) maintained in EMEM supplemented with 2% fetal bovine serum (Atlanta Biologicals). Confluent monolayers of HEp-2C cells in 24-well cell culture cluster plates (Costar) were inoculated with a 50 µL aliquot of EES suspension (four wells, at least 50 CCID_50_ per well) and incubated for three days at 33 °C, 5% CO_2_. Following two freeze-thaw cycles, cell lysates from the four wells were pooled, and virus isolates were harvested following removal of cell debris by centrifugation at 3000 x *g* for 10 min. The EES stool suspensions and virus isolates were assigned unique identifiers and shipped on dry ice to Viroclinics Biosciences B.V. (Rotterdam, the Netherlands) and stored frozen at −80 °C until analysis.

### Samples from nOPV2 phase 2 clinical trial

In the phase 2 trial (M5, NCT03554798), 49 or 51 healthy 1-to-5-year-old Panamanian participants primarily having received sequential schedules of bOPV and IPV were each orally administered at least one dose (approximately 10^6^ CCID_50_) of nOPV2-c1 or nOPV2-c2. The protocol was approved by the Ethical Review Committee of the Hospital del Niño 144 “Dr. José Renán Esquivel”. Written consent was obtained from parents or legal guardian(s) at enrollment per country regulations.

Stool samples were collected and shipped to CDC as in M2. Poliovirus genomes were detected and titrated as described above to identify EES for analysis by NGS and the modified mouse neurovirulence test. M2 and M5 EES were tested concomitantly in these assays, with analysts blinded to test article in the neurovirulence tests.

### Neurovirulence of shed virus

The WHO poliovirus receptor (PVR) transgenic mouse neurovirulence test (TgmNVT) used to release vaccine^24^ was modified (*m*TgmNVT) to assess the neurovirulence of shed virus from M2 and M5 clinical trials. The titers of Sabin OPV2 WHO international standard 15/296 (NIBSC) and virus isolates (from EES) were determined by CCID_50_ assay^23^ in Hep-2C cells at Viroclinics Biosciences B.V. prior to conducting the mTgmNVT. For each EES, ten 6-to-8-week-old Tg-PVR21 mice (CLEA/Japan) (5 of each sex) were randomized to receive intraspinal inoculations of 10^4^ CCID_50_ amplified virus (from EES) or control virus in 5 µl. Controls included 20 mice inoculated with Sabin OPV2 virus SO + 2/II at the 5.0 and 6.0 log_10_ CCID_50_ dose levels, as well as a sample of shed Sabin-2 virus collected day 7 post-mOPV2-challenge in a prior clinical trial^25^. Back-titrations of the diluted samples confirmed that titers were within 0.5 log_10_ CCID_50_ of the nominal dose. Inoculated mice were monitored for paralysis over a 14-day period per established protocol^24^.

When paralysis rates were ≥40%, the paralytic dose for 50% of mice (PD_50_) was determined. For these tests, groups of 10 Tg-PVR21 mice were inoculated with each dose and PD_50_ calculated using logistic regression.

The Tg-PVR21 mouse experiments at Viroclinics were conducted in compliance with Dutch Animal Testing Act (WOD) and according to the DIRECTIVE 2010/63/EU of The European Parliament and of the Council of 22 September 2010 on the protection of animals used for scientific purposes under project license 2770020171404 issued by the central authorizing body (Centrale Commissie Dierproven). Ethical approval for the study was obtained in working protocols 1-3 from the animal ethics committee (Instantie voor Dierenwelzijn) under the above project license.

### Statistical analysis

A binomial logistic generalized linear regression mixed model with mouse gender as a factor and subject (EES)-specific random effects was fitted with SAS version 9.4 to mouse paralysis data for each vaccine group separately, yielding estimates of paralysis rate. For comparison of paralysis rate of shed virus from M5 nOPV2-c1 and nOPV2-c2 participants versus M2 mOPV2, two additional similar models were fit which also included a term to estimate the odds ratio (OR) of paralysis for the virus shed from each candidate relative to shed mOPV2. This estimate is referred to as the adjusted odds ratio (aOR), as it is adjusted for mouse gender imbalance owing to mice excluded from evaluation due to reasons unrelated to the inoculum. Estimated PD_50_ values were obtained by inverting the model, with asymptotic confidence intervals computed via the delta method.

### Next-generation sequencing (NGS) of shed virus

NGS and data analysis were performed on viral RNA isolated from both cell-culture-amplified virus and from 10% stool suspensions of the EES of each participant, using a previously-described method^26,27^. The method was qualified and executed at Viroclinics Biosciences B.V. In brief, viral RNA was isolated from 140 µl amplified virus stock or stool suspension of each EES using QIAmp Viral RNA mini kit (Qiagen). cDNA preparation and amplification of full-length genome were performed as described elsewhere^19^. Tagmentation and library preparation by Illumina DNA Prep (M) Tagmentation were performed, followed by 300-cycle paired-end sequencing using MiSeq reagent kit v3 reagents on a MiSeq instrument with analysis software version 1.8.46 (Illumina) to generate FASTQ files. Data analysis was performed using the relevant candidate reference sequence (or to Sabin-2 reference sequence); Genbank accession number AY184220) and a proprietary algorithm at Viroclinics comprised of trimming (Trimgalore version 0.4.4), mapping (BWA mem; version 0.7.16a-r1181), and SNP (single nucleotide polymorphism) calling where multinucleotide polymorphisms and COMPLEXES are split into multiple SNPs using the vcfallelicprimitives utility from vcflib library (freebayes; version v1.1.0-60-gc15b070). Polymorphisms (present at ≥1%) were reported as SNPs with the impact on amino acid coding of the SNP noted per SNP. Re-analysis of select FASTQ files was conducted in Geneious Prime 2020.0.5 (Biomatters) to assess the possible co-location of VP1-143, VP1-N171D and VP1-E295K in nOPV2-c1 shed virus samples^16^. In brief, for these analyses, reads were merged and mapped against reference sequences from nucleotides 2969-3054 for 143/171 or 3052-3426 for 171/295, requiring the mapped reads to cover the full reference.

The criteria for reporting in Supplementary Tables 1, 3 and 5 are described below. Data provided in these Tables are represented in Figs. 2, 3 and 5 utilizing a color gradient scale. The mean frequency of a polymorphism of both stool replicates is represented along with the frequency of the polymorphism observed in the culture-amplified replicate for each EES. Where relevant, the figures also show the corresponding mTgmNVT result for each EES.

### Reporting polymorphisms in known attenuation sites and modified regions of shed viruses

Certain EES only had one or two evaluable components (replicates). For EES where both stool aliquots and culture-amplified virus isolate were evaluable, polymorphisms in known attenuation sites and modified regions of the strain present in at least two of the three replicates are indicated in the Supplementary Tables 1, 3 and 5. Similarly, if only two replicates of a sample (two stool or one stool and isolate) were available, and a SNP was present in both replicates, the change is indicated in the results tables. If a SNP was present in only 1 of 1, 1 of 2, or 1 of 3 available replicates (and thus not confirmed), it was only indicated in tables if present at ≥10% frequency. However, once a reporting threshold for a SNP was met, all positive results for other EES derived from that vaccine/candidate were tabulated in results for that study, even if only present in a single replicate.

All SNPs reported have Quality scores (Q scores) ≥ 30 unless otherwise indicated in the Supplementary Tables.

### Reporting polymorphisms in other regions of shed viruses

Mutations associated with amino acid changes present in other regions (outside of key attenuating and modified regions noted above) are provided in the Supplementary Tables 2, 4 and 6. If the mean frequency of a mutation associated with an amino acid change was 50% for an EES (regardless of the number of replicates available per EES), the change was tabulated. In cases where EES meet this criterion for a particular mutation, other EES showing the change were also tabulated even if present below 50%. If a fixed mutation (≥95%) associated with an amino acid change was observed in at least one of the replicates it was also tabulated.

### Reporting summary

Further information on research design is available in the Nature Research Reporting Summary linked to this article.

## Supplementary information

Supplementary Information

REPORTING SUMMARY

## Data Availability

Datasets specific to this publication are available from the corresponding author upon reasonable request and contingent on the requested uses being permitted under the informed consent received from the source clinical study, where applicable. The full genome sequence of nOPV2 candidate 1 can be found in GenBank (accession number MZ245455). The full genome sequence of nOPV2 candidate 2 can be found in GenBank (accession number MN654096). Sequencing data can be accessed on the SRA database (accession number PRJNA781433).

## Code availability

Algorithms for polymorphism selection is described under METHODS. R codes for tables and figures specific to this publication are available from the corresponding author upon reasonable request.
