# Supplementary material for: Evaluating stability of attenuated Sabin and two novel type 2 oral poliovirus vaccines in children
Source: NPJ Vaccines. 2022 Feb 11;7:19. doi: 10.1038/s41541-022-00437-5 (PMC8837630; doi:10.1038/s41541-022-00437-5)
Supplement: Supplementary file 1 — Supplementary Information [file 41541_2022_437_MOESM1_ESM.pdf]

Supplementary Information

a

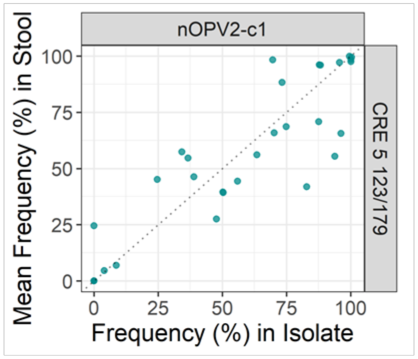

Virus	Mutation	Correlation coefficient
mOPV2	Domain IV U398C	1.000
mOPV2	VP1-I143X	0.896
nOPV2-c1	Cre5 123/179	0.892
nOPV2-c1	Domain IV U459C	0.577
nOPV2-c1	VP1-I143X	0.696
nOPV2-c2	Domain IV U398C	0.961
nOPV2-c2	VP1-I143X	0.800

b

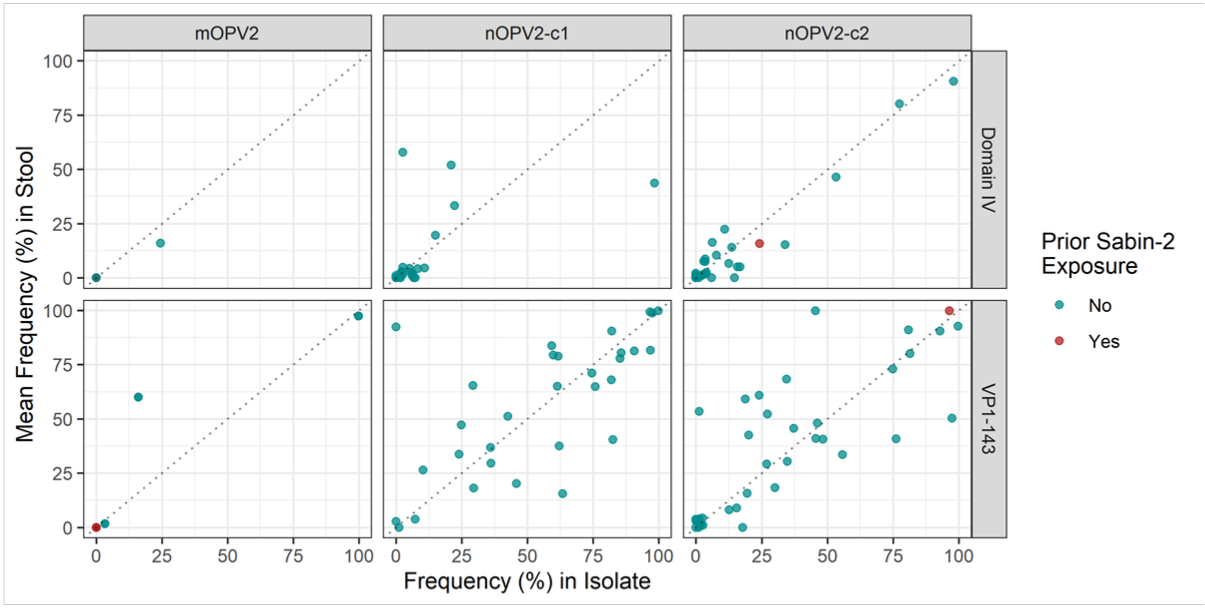

Supplementary Figure 1. Comparison of average frequency of selected polymorphims measured in EES stools and corresponding culture-amplified shed Sabin-2, nOPV2-c1 and nOPV2-c2 viruses. **a.** Cre5 123/179 **b**. Domain IV (nucleotide position 398 for Sabin-2/nOPV2-c2 and 456 in nOPV2-c1) and VP1-143. VP1-143 and cre5 123/179 polymorphisms presented in aggregate for each virus. The dotted diagonal line represents the identity (1:1) line. If SNP is only present in 1 stool that value is presented. If SNP is not present in either, assigned 0 for plotting purposes. All SNPs reported have Q scores ≥ 30. Pearson's correlation coefficients for each polymorphism in stool and culture-amplified isolate are shown.

Supplementary Table 1. *mOPV2.* Frequency of polymorphisms in EES at known attenuation sites

Genome unit			DomIV	DomV			VP1-143^			% Paralysis
	SNPs observed									
ID	*EES Day*	*Samples*	398(T->C)	481(A->G)	484(G->A)	489(T->C)	2908(A->G):VP1 143I>V	2909(T->C):VP1 143I>T	2909(T->A):VP1 143I>N	
M201003	4	SSI		0.01,0,0.1						0
M201033	5	SSI			0.02,0,0.06					0
M201030	7	SSI		0.85,0.91,0.98		0.03,0.01,0		0.03,0,0	0,0,0.03	90
M201006	21	SSI		1,0.97,1			0.05,0.04,0	0.91,0.95,1		90
M201018	21	SSI	0.32,0,0.25	1,1,1			0.02,0.59,0.01		0.59,0,0.15	80
M201053	28	I		1						n.a

EES day shown with stool 1, stool 2 and cell culture isolate (SSI), if present. SNPs and associated amino acid change indicated, if applicable. Blank cells = SNP not detected in stool or isolate; n.a.= Insufficient titre to test in mTgmNVT. ^NGS pipeline reports the variants as SNPs. Coding impact assumes changes are not in common genomes when changes in both 2908 and 2909 are seen.

Supplementary Table 2. *mOPV2.* Frequency of polymorphisms associated with amino acid changes in other regions of the genome

	SNPs observed		961(A->G):VP2 3N>D	1997(A->G):VP3 77H>R	% Paralysis
ID	*EES Day*	*Samples*			
M201003	4	SSI			0
M201033	5	SSI			0
M201030	7	SSI	0.3,0.35,0.25		90
M201006	21	SSI		0.58,0.41,0.51	90
M201018	21	SSI		0.9,0.67,0.94	80
M201053	28	I	0.58		n.a

EES day shown with stool 1, stool 2 and cell culture isolate (SSI), if present. SNPs and associated amino acid change indicated, if applicable. Blank cells = SNP not detected in stool or isolate; n.a.= insufficient titre to test in mTgmNVT.

Supplementary Table 3. *nOPV2-c1.* Frequency of polymorphisms in EES at known attenuation sites and modified regions of the candidate

	Genome unit		Cre5										DomIV	S15DomV			VP1-143^		2C cre KO					% Paralysis
iD	SNPs observed		121(C->T)	123(T->C)	131(T->C)	165(C->T)	171(C->CT)	172(T->A)	177(TAGTAC->T)	178(A->G)	179(G->A)	181(A->G)	459(T->C)	550(C->T)	566(A->G)	569(C->T)	2969(A->G): VP1 143I>V	2970(T->C): VP1 143I>T	4525(T->C)	4527(A->G): 2C 115K>R	4540(T->C)	4543(C->T)	4546(G->A)	
	EES Day	*Samples*																						
M5-3-123	2	SSI				0,0,0.04												0.2,0.2,0.46						
M5-3-150	3	SSI	0,0,0.04														0.03,0.02,0	0.25,0.23,0.1						
M5-3-149	4	SSI		0.49,0,0														0,0.31,0.63						0
M5-3-042	5	SSI																0.92,0.93,0						n.a
M5-3-043	5	SSI	0.03,0,0.01	0.64,0.52,0.55							0.16,0.44,0.18		0.05,0.03,0.05				0.04,0,0.04	0.66,0.73,0.7		0,0.02,0				0
M5-3-053	5	SSI		1,0.31,0.96														1,0.3,0.61						
M5-3-118	5	SSI		0.17,0,0.36							0.38,0,0.11		0,0,0.07					0.59,1,0.6						0
M5-3-148	5	SSI		0.98,0.89,0.88						0,0.01,0	0,0.04,0							0,0.08,0.07						0
M5-3-054	6	SSI		0.87,0.19,0.27							0,0.03,0.1		0,0.05,0.06					0.34,0.4,0.36						0
M5-3-059	6	SSI		0,0.05,0.04							0,0.04,0					0.06,0,0	0,0.01,0.03	0.78,0.84,0.94						0
M5-3-114	6	SSI		0.62,0.24,0.42							0.03,0,0.14		0,0,0.02					0.26,0.76,0.42			0.08,0,0.03	0,0,0.05		0
M5-3-154	6	SSI		0.83,0.48,0.7								0,0,0.02	0,0,0.02				0,0,0.02	0.41,0.18,0.34		0,0.02,0				0
M5-3-046	7	SSI	0,0,0.03	0.02,0.12,0.09		0,0.16,0.01							0.3,0.09,0.15					0.9,0.71,0.86						0
M5-3-060	7	SSI		0.33,0.33,0.66							0.09,0.09,0.17		0,0.04,0.02			0,0,0.02	0,0.06,0							0
M5-3-096	7	SSI		0.82,0.81,0.62							0.12,0.17,0.26		0,0.03,0.02	0,0.02,0.02	0,0,0.04		0,0.02,0	0.49,0.77,0.76						
M5-3-117	7	SSI	0,0.02,0	0.6,0.17,0.2					0,0.04,0.04		0,0.13,0.04		0,0.02,0					1,0.56,0.85	0.02,0,0					10
M5-3-119	7	SSI	0.02,0.01,0	0.12,0.31,0.21							0.49,0.2,0.43							0.62,0.74,0.82	0.08,0.15,0.03					
M5-3-070	8	SSI		0.29,0.3,0.19							0.28,0.27,0.15		0.04,0.04,0.08				0.03,0.03,0	0.34,0.27,0.24						0
M5-3-100	8	SSI															0.04,0.03,0	0.96,0.96,0.97						
M5-3-055	9	SI	0,0.02	0.33,0.86		0,0.05					0.22,0.07							0.4,0.83						
M5-3-076	9	SSI		0.23,0.05,0.25				0.53,0.21,0			0.22,0.29,0.25		0.53,0.63,0.03				0,0.25,0.02	0.94,0.62,0.8						
M5-3-151	9	SSI		0.22,0.17,0.24		0,0.02,0					0.78,0.8,0.45		0,0.02,0.06		0,0.04,0			0.78,0.8,0.62						0
M5-3-169	9	SSI		0.17,0.39,0.11							0.68,0.14,0.64		0.09,0,0.11		0,0,0.01		0.03,0,0.08	0.82,0.78,0.83		0,0.19,0				0
M5-3-081	10	SSI		0.77,0.75,0.81							0.23,0.24,0.19		0.02,0.01,0.06		0,0,0.01		0,0,0.01							0
M5-3-083	10	SSI		1,0.66,0.76							0,0.29,0.2		0,0.1,0.03					0.89,0.78,0.59						0
M5-3-124	10	SSI		0.5,0.33,0.73							0.19,0.4,0.15		0.06,0,0.02				0.13,0.13,0.12	0.49,0.56,0.17						0
M5-3-128	10	SSI		0.15,0.66,0.16	0.07,0.22,0.02						0,0.12,0.23		0,0,0.07					0.29,0.65,0.25					0.1,0,0	0
M5-3-157	10	SSI		0.58,0.82,0.83							0.38,0.18,0.17	0.03,0,0.01	0.25,0.41,0.22			0.02,0,0.1	0,0.02,0	0.55,0.15,0.62			0.01,0,0		0.02,0,0	0
M5-3-164	10	SSI		0.34,0.39,0.44							0.06,0,0.06		0.03,0,0.03					0.29,0.07,0.3						0
M5-3-050	15	SSI		0.89,1,0.99							0.06,0,0.01	0.05,0,0.02	0.59,0.45,0.21		0.01,0,0.04			0.98,1,0.98						0
M5-3-162	28	SSI		1,0,1			0.18,0,0			0.26,0,0.11	0,1,0		0.87,0,0.98					1,1,1				0.09,0,0.02		11.11

No variants observed at 3Dpol-38 or 3D-pol-53 modifications. EES day shown with stool 1, stool 2 and cell culture isolate (SSI), if present. SNP and associated amino acid change indicated, if applicable. Paralysis rates are indicated for the samples that have mTgmNVT results available. Blank cell = variant not detected in stool or isolate; Gray cell = result not available; n.a. = insufficient titre to test in mTgmNVT.^NGS pipeline reports the variants as SNPs. Coding impact assumes changes are not in common genomes when changes in both 2969 and 2970 are seen.

Supplementary Table 4. *nOPV2-c1.* Frequency of polymorphisms associated with amino acid changes in other regions of the genome

	SNPs observed		2058(A->G):VP3 77H>R	2060(T->C):VP3 78S>P	2528(G->A):VP3 234E>K	3053(A->G):VP1 171N>D	3425(G->A):VP1 295E>K	5645(G->A):3C-pol 50D>N	% Paralysis
ID	*EES_day*	*Samples*							
M5-3-123	2	SSI			0.21,0.25,0.43	0,0.02,0.33	0,0.05,0.33		
M5-3-150	3	SSI			0.32,0.31,0.11	0,0.04,0.46	0,0.04,0.46		
M5-3-149	4	SSI			0.51,0.1,0.08				0
M5-3-042	5	SSI			0.98,0.95,1	0.05,0.02,1			n.a
M5-3-043	5	SSI			0.12,0.2,0.11	0.01,0.02,0.01		0,0.02,0	0
M5-3-053	5	SSI			1,0.32,0.52				
M5-3-118	5	SSI			0,0,0.1	0,0,0.01			0
M5-3-148	5	SSI			0.02,0.08,0.03	0.02,0,0			0
M5-3-054	6	SSI			0.35,0.29,0.09				0
M5-3-059	6	SSI			0.06,0.03,0				0
M5-3-114	6	SSI			0.28,0.78,0.43	0,0,0.04			0
M5-3-154	6	SSI			0.19,0.17,0.22			0,0,0.03	0
M5-3-046	7	SSI			0.05,0,0.06	0.05,0,0.02		0.03,0,0.01	0
M5-3-060	7	SSI				0.08,0,0.03	0.08,0,0.02		0
M5-3-096	7	SSI			0,0.01,0				
M5-3-117	7	SSI			1,0.37,0.56	0,0,0.06	0,0,0.06	0,0,0.04	10
M5-3-119	7	SSI			0,0.04,0.01			0.04,0,0	
M5-3-070	8	SSI			0.03,0.03,0				0
M5-3-100	8	SSI			0.03,0.06,0.11				
M5-3-055	9	SI			0.4,0.42				
M5-3-076	9	SSI			0.16,0.18,0.19	0.05,0.07,0.06			
M5-3-151	9	SSI				0,0,0.2	0,0,0.18	0,0.03,0	0
M5-3-169	9	SSI	0,0.01,0		0.07,0.18,0.06				0
M5-3-081	10	SSI						0,0.01,0	0
M5-3-083	10	SSI			1,0.74,0.6	0.1,0,0.06			0
M5-3-124	10	SSI			0.3,0.58,0.1	0.31,0.22,0.65	0.3,0.16,0.65		0
M5-3-128	10	SSI				0.85,0.33,0.75	0.85,0.22,0.52		0
M5-3-157	10	SSI			0.51,0.17,0.6	0.06,0.13,0.12	0.04,0.12,0.12		0
M5-3-164	10	SSI			0.3,0.08,0.29				0
M5-3-050	15	SSI	0.2,0,0.81		0.8,1,0.16	0.05,0,0.06			0
M5-3-162	28	SSI	0.95,0.98,0.89	0,0.98,0				0.57,1,0.66	11.11

EES day shown with stool 1, stool 2 and cell culture isolate (SSI), if present. SNPs and associated amino acid change indicated, if applicable. Blank cell = variant not detected in stool or isolate; Gray cell = result not available; n.a = insufficient titre to test in mTgmNVT

Supplementary Table 5. *nOPV2-c2.* Frequency of polymorphisms in EES at known attenuation sites and modified regions of the candidate

	Genome unit		DomIV	S15domV					VP1-143^				
ID	SNPs observed		398(T->C)	482(C->T)	486(C->T)	489(C->T)	495(C->T)	505(A->G)	2908(A->G):VP1 143I>V	2909(T->C):VP1 143I>T	2909(T->G):VP1 143I>S	2909(T->A):VP1 143I>N	% Paralysis
	EES Day	*Samples*											
M5-3-061	1	S	0.01,0,0						1,0,0	0,0.07,0.01			n.a
M5-3-084	1	SSI											
M5-3-098	2	SSI								0.02,0.02,0.01			0
M5-3-101	2	SSI							0.06,0,0				0
M5-3-147	2	SSI							0.02,0,0	0,0,0.03			10
M5-3-167	2	SSI			0,0,0.03								
M5-3-048	3	SSI			0.02,0,0					0.03,0.02,0.01			10
M5-3-156	4	SSI							0.07,0,0	0.62,0.68,0.34			30
M5-3-077	5	SSI	0.48,0.45,0.53						0.42,0.34,0.42	0.03,0.03,0.04	0,0.01,0		
M5-3-106	5	SSI	0,0,0.15				0.13,0,0			0.33,0.26,0.27			0
M5-3-135	5	SSI	0.01,0,0							0.09,0,0.03			
M5-3-159	5	SSI			0,0.35,0				0.01,0,0	0.17,0,0.16			0
M5-3-057	6	SSI	0.06,0.04,0.16						0,0,0.01				0
M5-3-069	6	SSI	0,0,0.06						0,0,0.05	0,0.31,0.14			0
M5-3-087	6	SSI	0.1,0.05,0.04						0.02,0.02,0.02	0.31,0.25,0.33			0
M5-3-089	6	SSI	0,0.02,0.02			0.07,0.03,0.02				0,0.02,0.02			
M5-3-107	6	SSI	0,0.02,0.02		0.58,0.78,0.73		0,0,0.02			0.73,0.73,0.75			30
M5-3-130	6	SSI	0,0.05,0.04		0,0.03,0					1,0.18,0.19			0
M5-3-138	6	SSI	0,0.13,0.13		0.04,0,0		0,0,0.01			0.78,0.82,0.81			40
M5-3-071	7	SSI	0,0.03,0.04							0,0.02,0	0,0.05,0		
M5-3-090	7	SSI	0,0.01,0							0.48,0.33,0.48			
M5-3-047	8	SSI	0.03,0,0.03						0.01,0,0	0.4,0.36,0.76	0.01,0,0	0.03,0,0	30
M5-3-068	8	SSI	0,0.45,0.11	0.12,0,0	0,0,0.03					0,0.08,0.01			40
M5-3-132	8	SSI	0.22,0.09,0.34						0.05,0,0	0.27,0.72,0.23			10
M5-3-158	8	SSI							0,0.54,0.22	1,0.45,0.24			0
M5-3-045	9	SSI	0.11,0.1,0.08		0,0,0.02	0,0,0.06		0,0.02,0.01		0.1,0.27,0.3			40
M5-3-095	9	SSI	0.21,0.11,0.06				0.24,0.4,0.25			0.28,0.5,0.14		0.08,0,0.01	20
M5-3-121	9	SSI	0,0.15,0.03						0,0,0.1	0.41,0.26,0.46			
M5-3-044	10	SSI	0.16,0.12,0.14		0,0.01,0		0.08,0,0.01	0,0,0.16	0,0.08,0	0.48,0.44,0.98			50
M5-3-052	10	SI	0.04,0,0							1,0.86,1			0
M5-3-066	10	SSI	0.02,0,0					0,0,0.03	0.01,0,0	0.65,0.56,0.24			40
M5-3-131	10	SSI							0,0.02	0.91,0.77			0
M5-3-140	10	SI	0,0.01						0,0.08	0,0.1			0
M5-3-082	14	SSI	0.85,0.96,0.98						0.07,0.11,0	0.38,0.32,0.36	0.04,0,0.01		80
M5-3-086	14	SSI	0.1,0.08,0.04		0,0.03,0			0,0.06,0	0.06,0,0.01	0.07,0.04,0.11			10

	Genome unit		DomIV	S15domV					VP1-143^				
ID	SNPs observed		398(T->C)	482(C->T)	486(C->T)	489(C->T)	495(C->T)	505(A->G)	2908(A->G):VP1 143I>V	2909(T->C):VP1 143I>T	2909(T->G):VP1 143I>S	2909(T->A):VP1 143I>N	% Paralysis
	EES Day	*Samples*											
M5-3-145	14	SSI	0.32,0,0.24		0.01,0,0			0,0,0.09	0,0,0.1	1,1,0.86			10
M5-3-085	21	SSI	0.1,0,0.17			0.01,0,0		0.01,0,0		0.38,0.43,0.46	0.09,0,0	0.06,0,0	20
M5-3-170	21	SSI	0.6,1,0.78							0.81,1,0.93			30

EES day shown with stool 1, stool 2 and cell culture isolate (SSI). SNPs and associated amino acid change indicated, if applicable. Paralysis rates are indicated for the samples that have mTgmNVT results available. EES flagged for PD50 testing if paralysis ≥40%. Gray cells = result not available; Blank cells = variant not detected in stool or isolate; n.a = Isolate not available for testing. ^NGS pipeline reports the variants as SNPs. Coding impact assumes changes are not in common genomes when changes in both 2908 and 2909 are seen.

Supplementary Table 6. *nOPV2-c2.* Frequency of polymorphisms associated with amino acid changes in other regions of the genome

	SNPs observed		805(T->C): VP4 20Y>H	869(C->T): VP4 41A>V	1385(A->G): VP2 144Y>C	1997(A->G): VP3 77H>R	2401(G->A): VP3 212G>S	3577(G->A): 2A 65E>K	3900(T->A): 2B 23D>E	4870(G->A): 2C 250V>I	5251(A->G): 3A 48S>G	5584(G->A): 3C-pol 50D>N	5615(A->G): 3C-pol 60K>R	6386(A->G): 3D-pol 134Q>R	6643(A->G): 3D-pol 220K>E	% Paralysis
**ID**	*EES Day*	*Samples*														
M5-3-061	1	S		0,0,0.1	1,0,0		0,0.02,0			1,0,0						n.a
M5-3-084	1	SSI						1								
M5-3-098	2	SSI		0.04,0.06,0.07		0.02,0.01,0	0.03,0.04,0.02	0,0.01,0								0
M5-3-101	2	SSI		0,0.02,0.05			0.06,0.04,0									0
M5-3-147	2	SSI		0.12,0.01,0.07			0.02,0.06,0.02									10
M5-3-167	2	SSI		0.07,0.02,0.08		0.01,0,0.01	0.15,0.08,0.03					0,0,0.02		0.1,0,0		
M5-3-048	3	SSI		0,0.03,0.15			0.05,0.05,0									10
M5-3-156	4	SSI		0,0.02,0		0,0.01,0	0.04,0.06,0.01									30
M5-3-077	5	SSI		0,0.02,0.03			0.01,0,0									
M5-3-106	5	SSI		0,0,0.2		0,0,0.02	0,0.14,0						0.01,0,0			0
M5-3-135	5	SSI										0,0.11,0.11				
M5-3-159	5	SSI		0.13,0,0.14		0.14,0,0.12	0.08,0,0.06									0
M5-3-057	6	SSI		0.11,0.07,0.37		0,0,0.16	0.12,0.17,0.08									0
M5-3-069	6	SSI					0,0,0.01							1,0,0		0
M5-3-087	6	SSI	0,0.02,0	0.01,0.02,0.02		0.01,0,0	0.04,0.07,0.02									0
M5-3-089	6	SSI					0,0.03,0.01									
M5-3-107	6	SSI		0.1,0,0.05			0,0.02,0									30
M5-3-130	6	SSI	1,0,0	0,0,0.04			0,0.03,0									0
M5-3-138	6	SSI		0.03,0,0			0.06,0.01,0.02					0,0.01,0				40
M5-3-071	7	SSI														
M5-3-090	7	SSI					0.17,0.03,0									
M5-3-047	8	SSI		0.45,0.47,0.73		0.01,0,0	0.06,0.09,0									30
M5-3-068	8	SSI		0,0,0.02												40
M5-3-132	8	SSI		0,0,0.12			0.05,0,0.03					0.05,0,0				10
M5-3-158	8	SSI					0,0,0.08				1,0,0					0
M5-3-045	9	SSI					0.16,0.23,0.21							0,0.03,0		40
M5-3-095	9	SSI		0.07,0,0.03		0,0,0.06	0.06,0.02,0					0.06,0.02,0				20
M5-3-121	9	SSI		0,0,0.01			0,0,0.18									
M5-3-044	10	SSI					0.44,0.24,0.48									50
M5-3-052	10	SI					0.99,0.99,0.99			0,0,0.03						0
M5-3-066	10	SSI					0.06,0.26,0.11									40
M5-3-131	10	SSI					0,0.03									0

	SNPs observed		805(T->C): VP4 20Y>H	869(C->T): VP4 41A>V	1385(A->G): VP2 144Y>C	1997(A->G): VP3 77H>R	2401(G->A): VP3 212G>S	3577(G->A): 2A 65E>K	3900(T->A): 2B 23D>E	4870(G->A): 2C 250V>I	5251(A->G): 3A 48S>G	5584(G->A): 3C-pol 50D>N	5615(A->G): 3C-pol 60K>R	6386(A->G): 3D-pol 134Q>R	6643(A->G): 3D-pol 220K>E	% Paralysis
**ID**	*EES Day*	*Samples*														
M5-3-140	10	SI		1,0.65		1,0.8	0,0.04		1,0							0
M5-3-082	14	SSI										0.45,0.38,0.37				80
M5-3-086	14	SSI					0.82,0.89,0.8					0.81,0.89,0.77				10
M5-3-145	14	SSI										0,0,0.02				10
M5-3-085	21	SSI														20
M5-3-170	21	SSI				0.16,0,0.06							0.61,1,0.79	0,0,0.03	0.58,1,0.78	30

EES day shown with stool 1, stool 2 and cell culture isolate (SSI). SNPs and associated amino acid change indicated, if applicable. Gray cells = result not available; Blank cells = variant not detected in stool or isolate; n.a = Isolate not available for testing.
